# A synthetic lethal screen identifies ATR-inhibition as a novel therapeutic approach for POLD1-deficient cancers

**DOI:** 10.18632/oncotarget.6857

**Published:** 2016-01-09

**Authors:** Sandra Hocke, Yang Guo, Albert Job, Michael Orth, Andreas Ziesch, Kirsten Lauber, Enrico N De Toni, Thomas M. Gress, Andreas Herbst, Burkhard Göke, Eike Gallmeier

**Affiliations:** ^1^ Department of Medicine II, Ludwig-Maximilians-University of Munich, 81377 Munich, Germany; ^2^ Department of Gastroenterology, Endocrinology and Metabolism, University Hospital of Marburg, Philipps-University of Marburg, 35043 Marburg, Germany; ^3^ Department of Radiotherapy and Radiation Oncology, Ludwig-Maximilians-University of Munich, 81377 Munich, Germany

**Keywords:** ATR, POLD1, synthetic lethality, DNA repair, targeted therapy

## Abstract

The phosphoinositide 3-kinase-related kinase ATR represents a central checkpoint regulator and mediator of DNA-repair. Its inhibition selectively eliminates certain subsets of cancer cells in various tumor types, but the underlying genetic determinants remain enigmatic. Here, we applied a synthetic lethal screen directed against 288 DNA-repair genes using the well-defined *ATR* knock-in model of DLD1 colorectal cancer cells to identify potential DNA-repair defects mediating these effects. We identified a set of DNA-repair proteins, whose knockdown selectively killed *ATR*-deficient cancer cells. From this set, we further investigated the profound synthetic lethal interaction between *ATR* and *POLD1*. *ATR*-dependent *POLD1* knockdown-induced cell killing was reproducible pharmacologically in POLD1-depleted DLD1 cells and a panel of other colorectal cancer cell lines by using chemical inhibitors of ATR or its major effector kinase CHK1. Mechanistically, POLD1 depletion in *ATR*-deficient cells caused caspase-dependent apoptosis without preceding cell cycle arrest and increased DNA-damage along with impaired DNA-repair. Our data could have clinical implications regarding tumor genotype-based cancer therapy, as inactivating *POLD1* mutations have recently been identified in small subsets of colorectal and endometrial cancers. *POLD1* deficiency might thus represent a predictive marker for treatment response towards ATR- or CHK1-inhibitors that are currently tested in clinical trials.

## INTRODUCTION

Genome integrity is ensured by a complex DNA damage response (DDR) network. Alterations in this network, predisposing cells to exogenous and endogenous genotoxic stress, are often linked to tumorigenesis [1, 2] and compensatory DNA-repair gene activation [2]. Some DNA-repair pathways might thus provide exploitable targets through synthetic lethal interactions in subgroups of tumors harboring certain DNA-repair defects and thus facilitate novel selective and tumor-specific therapeutic approaches besides the classical chemo- and radiotherapeutic regimens.

Synthetic lethality is defined as the interaction of two non-lethal mutations, which in combination are incompatible with cell viability. This mechanism could facilitate tumor-specificity for pharmacologic therapeutic approaches through the specific targeting of defined tumor cell alterations with agents, which act synthetically lethal with these alterations [3]. Consequently, a weak single-agent anticancer activity could be potentiated in certain subpopulations of tumor patients [4]. One of the most striking examples for this approach is illustrated by the inhibition of PARP in *BRCA1*- and *BRCA2*-deficient cancers and is currently under intense clinical investigation [5, 6]. In addition, several other synthetic lethal interactions between DDR genes elicited by either classical gene knockout or chemical inhibition have been reported [7–9].

The phosphoinositide 3-kinase (PIK)-related kinase ATR is a central regulator of the replication checkpoint during DDR signaling [10]. At sites of replication stress or DNA damage, ATR promotes cell cycle arrest induction and replication fork stabilization prior to the initiation of homologous recombination-mediated DNA-repair [11, 12]. Recently, several compounds for the specific targeting of ATR have been developed [13]. These ATR-inhibitors cause the elimination of certain subsets of tumor cells, but the underlying mechanisms remain poorly defined [14, 15]. Due to the central role of ATR in the DDR, synthetic lethal interactions of ATR with certain tumor-mutated DNA-repair genes might at least partly explain this selective tumor cell killing by ATR-inhibitors. In fact, pharmacological inhibition of ATR has previously been demonstrated to act synthetically lethal with *ATM*, *XRCC1* and *ERCC1* deficiency [16–19] as well as *CYCLIN E* and oncogenic *RAS* overexpression [20, 21].

The aim of this study was to identify synthetically lethal interactions between *ATR* and certain DNA-repair genes, applying a siRNA library of all major DNA-repair genes in a well-characterized genetic *ATR* knock-in model of DLD1 colorectal cancer (CRC) cells [14, 22, 23] harboring the hypomorphic *ATR*-Seckel mutation. From the identified set of DNA-repair genes that act synthetically lethal with *ATR*, the profound effects of *POLD1* were further characterized.

## RESULTS

### siRNA library screening to identify synthetic lethal interactions between ATR and DNA-repair genes in DLD1 cells

**Figure 1:**
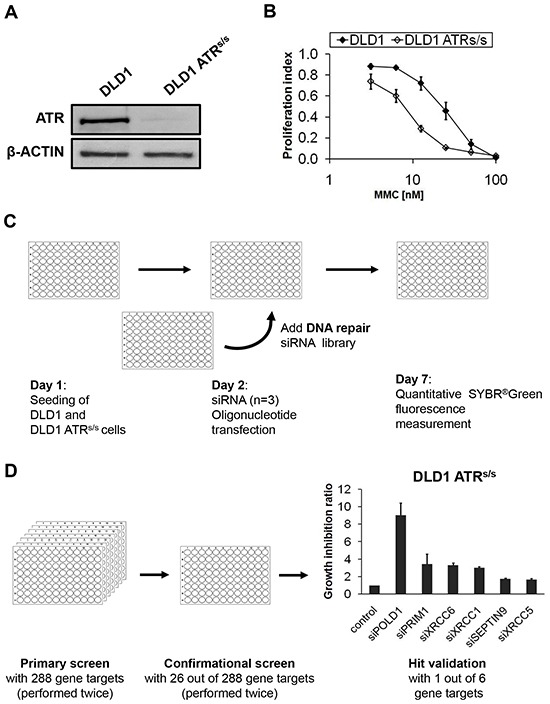
Experimental design and screening process of the siRNA library screening **A.** ATR protein synthesis was assessed in parental and *ATR^s/s^* cells by immunoblotting. β-ACTIN served as loading control. **B.** MMC sensitivity of parental and *ATR*^s/s^ cells was assessed at 120 h after treatment by proliferation assay. Error bars represent SEM of three independent experiments with each data point reflecting triplicate wells. **C.** Experimental procedure of the siRNA library screening. **D.** Multiple siRNA screens gradually identified the top six candidate genes exhibiting synthetic lethal interactions with *ATR*. Error bars represent SEM of four independent experiments.

To identify potential synthetically lethal interactions between *ATR* and certain DNA-repair genes, we compared the effects of siRNA-mediated knockdown of single genes on the proliferation rate of *ATR*-proficient parental versus *ATR*-deficient *ATR^s/s^* DLD1 cancer cells harboring the *ATR* knock-in Seckel mutation [23], using a focused siRNA library directed against 288 DNA repair genes each targeted by three different siRNAs. Prior to screening, *ATR* deficiency of *ATR^s/s^* cells was verified on the protein level by demonstration of ATR protein suppression below the detection limit of our assay (Figure 1A) and functionally through confirmation of hypersensitivity towards the DNA interstrand-crosslinking (ICL) agent mitomycin C (MMC) (Figure 1B) [24, 25]. The experimental screening design is schematically outlined in Figure 1C and Figure 1D. In short, parental and *ATR^s/s^* cells were transfected simultaneously using a previously established siRNA library. At 120 h post transfection, proliferation differences between *ATR-*proficient and -deficient cells were assessed. This primary screen was independently performed twice and generated 26 primary hits (9%), which were again verified twice in the conformational screen and classified into hit categories as selective *ATR* genotype-dependent and *ATR* genotype-independent proliferation inhibition, respectively, according to the criteria described in the Material&Methods section. Taken together, each candidate gene was validated based on the average growth inhibition ratio of four independent experiments. The top six gene targets displaying selective *ATR*-genotype dependent proliferation inhibition are summarized in Figure 1D and Table 1. The strongest effect was observed for *POLD1* (9-fold growth inhibition ratio with an average relative survival of 5% of *ATR^s/s^* cells) and therefore chosen for further in-depth characterization.

**Table 1:** Identified *ATR* genotype-dependent DNA-repair gene targets

Rank	Gene Target	Growth Inhibition Ratio*	Average Relative Survival DLD1	Average Relative Survival DLD1 *ATR^s/s^*
1	***POLD1***	9.04±1.42	0.47	0.05
2	***PRIM1***	3.43±1.15	0.47	0.17
3	***XRCC6 (Ku70)***	3.34±0.23	0.68	0.30
4	***XRCC1***	3.03±0.12	0.60	0.20
5	***SEPT9***	1.74±0.11	0.73	0.42
6	***XRCC5 (Ku80)***	1.66±0.12	0.64	0.38

* The growth inhibition ratio was calculated by dividing the growth inhibition value of parental by the value of *ATR^s/s^* cells. The mean growth inhibition ratio and SEM were determined from four individual growth inhibition ratio values that each represent triplicates from three different oligonucleotides targeting one particular gene, as described in Material&Methods.

### *ATR*-genotype independent gene knockdown-induced detrimental effects on DLD1 cells

In addition to the identified synthetic lethal interactions, siRNA-mediated knockdown of 20 genes induced detrimental effects in DLD1 cells independent of *ATR*-genotype (average relative survival between 6% and 35% in parental and *ATR^s/s^* cells) (Table 2). Notably, siRNA-mediated knockdown of *XAB2* and *PLK1* caused a virtually complete loss of proliferation, extending the known essential functions of these genes also to DLD1 colorectal cancer cells [26, 27].

**Table 2:** Identified *ATR* genotype-independent DNA-repair gene targets

Rank	Gene Target	Growth Inhibition Ratio*	Average Relative Survival DLD1	Average Relative Survival DLD1 *ATR^s/s^*	Average relative survival DLD1 and DLD1 ATR^s/s^**
1	***XAB2***	1.40±0.46	0.06	0.05	0.06
2	***PLK1***	2.51±1.86	0.12	0.03	0.08
3	***RPL35***	0.58±0.17	0.07	0.14	0.11
4	***PSMC4***	1.73±1.14	0.16	0.11	0.14
5	***RPL27***	0.21±0.07	0.04	0.23	0.14
6	***NUP205***	2.85±2.29	0.18	0.15	0.17
7	***RRM1***	1.75±1.04	0.22	0.11	0.17
8	***POLE***	1.63±0.80	0.22	0.12	0.17
9	***RRM2***	1.40±0.39	0.23	0.15	0.19
10	***PSMA1***	0.61±0.24	0.27	0.11	0.19
11	***POLA1***	1.66±1.13	0.22	0.18	0.20
12	***RPA2***	1.68±0.32	0.26	0.15	0.21
13	***RPA1***	0.93±0.34	0.22	0.21	0.22
14	***SNRPF***	1.06±0.63	0.23	0.21	0.22
15	***ENDOV***	0.74±0.10	0.24	0.35	0.30
16	***FBXO18***	0.85±0.21	0.27	0.35	0.31
17	***PMS2P5***	1.66±1.02	0.41	0.20	0.31
18	***PARP4***	1.60±0.62	0.40	0.23	0.32
19	***FEN1***	0.70±0.17	0.28	0.41	0.35
20	***PCNA***	1.83±1.00	0.45	0.25	0.35

* The growth inhibition ratio was calculated by dividing the growth inhibition value of parental DLD1 by the value of *ATR^s/s^* cells. The mean growth inhibition ratio and SEM were determined from four individual growth inhibition ratio values that each represent triplicates from three different oligonucleotides targeting one particular gene.

** The average relative survival of parental and ATRs/s cells, respectively, was calculated by the mean of four individual growth inhibition values for each cell line from three different oligonucleotides targeting one particular gene, as described in Material&Methods.

### Validation of synthetic lethality of *ATR* with *POLD1* in *ATR^s/s^* cells

**Figure 2:**
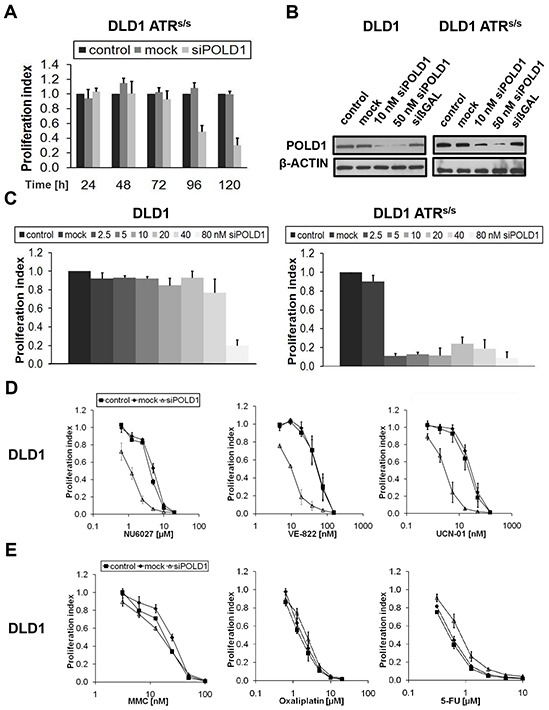
ATR-/CHK1-dependent proliferation inhibition upon *POLD1* knockdown in DLD1 cancer cells **A.** Proliferation inhibition over time of siRNA-mediated *POLD1* knockdown (10 nM) was assessed in *ATR^s/s^* cells. **B.** Efficient siRNA-mediated POLD1 protein depletion was confirmed at 96 h after treatment in parental and *ATR^s/s^* cells. siβGAL served as transfection control, β-ACTIN as loading control. **C.** *siPOLD1* concentration-dependent proliferation inhibition was assessed at 120 h after treatment in parental and *ATR^s/s^* cells. **D+E.** Effects on proliferation of ATR- and CHK1-inhibitors (D) or common chemotherapeutics (E), respectively, were assessed at 120 h after treatment in control-, mock- or *siPOLD1*-treated DLD1 cells. Error bars represent SEM of three independent experiments with each data point reflecting triplicate wells.

To validate the synthetic lethal relationship of *ATR* with *POLD1*, time- and dose-kinetics were performed upon siRNA-mediated POLD1 depletion in *ATR^s/s^* cells. The detrimental effects of *POLD1* knockdown selectively on *ATR^s/s^* cells were time-dependent, as shown by a proliferation inhibition of at least 50%, starting at 96 h and further peaking at 120 h post transfection, as compared to mock- and untreated *ATR^s/s^* cells (Figure 2A). Efficient siRNA-mediated *POLD1* knockdown at 96 h post transfection was confirmed on the protein level in parental and *ATR^s/s^* cells (Figure 2B). Similarly, the effects of *POLD1* knockdown on *ATR^s/s^* cells were dose-dependent, as shown at 120 h post transfection by a proliferation inhibition of at least 70% at concentrations ranging from 2.5 nM to 40 nM (Figure 2C). Expectedly, *ATR*-genotype independent proliferation inhibition was observed in parental and *ATR^s/s^* cells upon *siPOLD1* treatment at higher and likely toxic siRNA concentrations starting from 80 nM. Importantly, clonally selected heterozygous *ATR^+/s^* cells also remained unaffected by *POLD1*-knockdown, excluding artefacts due to clonal variability (data not shown).

### *siPOLD1*-mediated sensitization of DLD1 cells towards ATR- and CHK1-inhibitors

To test whether the *siPOLD1*-mediated effects on *ATR*-deficient DLD1 cells were reproducible through chemical inhibition of ATR or its major downstream effector kinase CHK1 in *ATR*-proficient DLD1 cells, the ATR-inhibitors NU6027 and VE-822 as well as the rather unselective but only currently FDA-approved CHK1-inhibitor UCN-01 were applied. A significantly increased sensitivity towards NU6027 (IC50 ratio 4), VE-822 (IC50 ratio 5) and UCN-01 (IC50 ratio 8) was observed at 120 h selectively in POLD1-depleted as compared to control or mock-transfected DLD1 cells (Figure 2D). To exclude a general unspecific hypersensitivity phenotype, POLD1-depleted and control DLD1 cells were treated with commonly used chemotherapeutics including ICL- and non-ICL-agents (MMC, oxaliplatin, 5-fluorouracile (5-FU)). No significant proliferation differences among POLD1-depleted, mock-transfected and control cells were detected upon treatment with any of these agents (Figure 2E).

### Generalization of siPOLD1-mediated sensitization towards ATR- and CHK1-inhibitors using a panel of colorectal cancer cell lines

**Figure 3:**
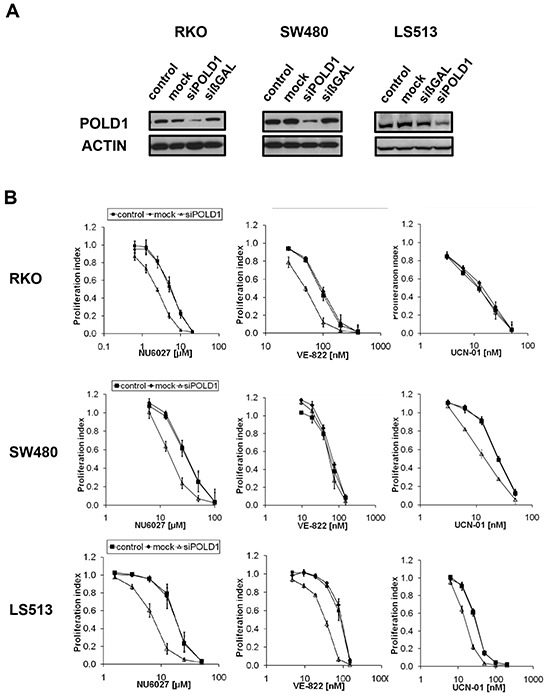
ATR-/CHK1-dependent proliferation inhibition upon *POLD1* knockdown in a panel of CRC cell lines **A.** Efficient siRNA-mediated POLD1 protein depletion was confirmed at 96 h after treatment in RKO, SW480 and LS513 cells. β-ACTIN served as loading control. **B.** Effects on proliferation of ATR- and CHK1-inhibitors were assessed at 120 h after treatment in control-, mock- or *siPOLD1*-treated RKO, SW480 and LS513 cells. Error bars represent SEM of three independent experiments with each data point reflecting triplicate wells.

In an effort to generalize our data beyond one single cell line, we applied a panel of CRC cell lines. After optimization and confirmation of efficient *POLD1* knockdown for each line (Figure 3A), the cells were treated with NU6027, VE-822 or UCN-01, respectively. As compared to control cells, POLD1 depletion sensitized RKO cells towards NU6027 (IC50 ratio 3) and VE-822 (IC50 ratio 2) (Figure 3B, upper panel), SW480 cells towards NU6027 (IC50 ratio 2) and UCN-01 (IC50 ratio 2) (Figure 3B, middle panel) and LS513 cells towards all three inhibitors tested (IC50 ratio 2-3) (Figure 3B, lower panel). To exclude a general unspecific hypersensitivity phenotype, POLD1-depleted, mock-transfected and control RKO, SW480 and LS513 cells were treated with MMC, oxaliplatin or 5-FU, respectively. No significant differences in proliferation rates were detected among POLD1-depleted, mock-transfected and control cells upon treatment with any of these agents (Supplementary Figure S1).

### *siPOLD1*-mediated apoptosis in *ATR^s/s^* cancer cells

**Figure 4:**
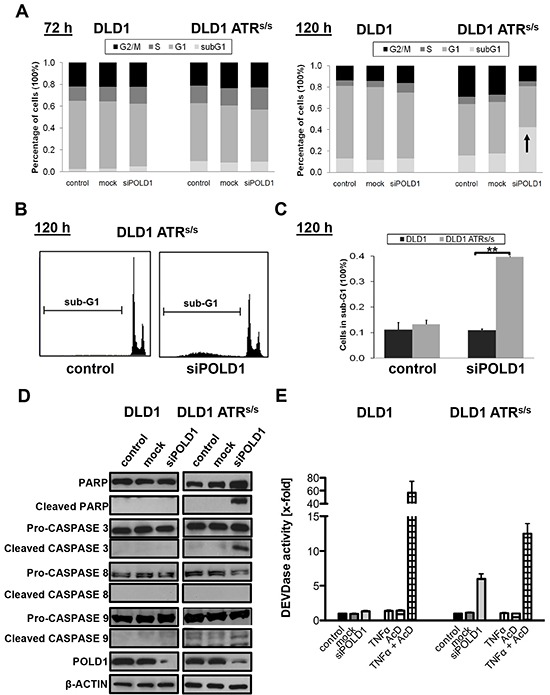
*ATR* genotype-dependent effects of POLD1 depletion on cell cycle profile and apoptosis Cell cycle and apoptosis analyses were performed upon siRNA-mediated *POLD1* knockdown at 10 nM in parental and *ATR^s/s^* cells. **A.** Representative cell cycle profiles at 72 h and 120 h after siRNA-treatment, **B.** representative histograms of sub-G1 fractions from one experiment at 120 h after treatment and **C.** statistical analysis of sub-G1 fractions from three independent experiments at 120 h after treatment are shown for parental and *ATR^s/s^* cells. Error bars represent SEM of three independent experiments. Asterisks mark statistical significance between two samples using the Student's t-test (**p < 0.01). **D.** Cleavage of PARP, CASPASE3 and CASPASE9 upon POLD1 depletion at 96 h after siRNA treatment in *ATR^s/s^* cells. β-ACTIN served as loading control. **E.** Fluorometric analysis of intracellular CASPASE3-mediated DEVDase activity was analyzed at 96 h after siRNA treatment. Combinational treatment with TNFα (25 ng/ml) and AcD (200 ng/ml) served as positive control for CASPASE3 activity. Error bars represent SEM of two experiments, independently performed in triplicates.

We next analyzed the mechanism underlying *POLD1* knockdown-mediated cell killing of *ATR*-deficient cells. Cell cycle distribution and sub-G1 fraction were assessed upon *siPOLD1* transfection at 10 nM in parental versus *ATR*^s/s^ cells. No significant baseline differences in cell cycle profiles or sub-G1 content were detected among control-, mock- or *siPOLD1*-transfected cells at 72 h (Figure 4A). In contrast, *ATR^s/s^* but not parental DLD1 cells displayed a slightly increased sub-G1 fraction at 96 h after *siPOLD1-*transfection (10%, data not shown), which strongly increased at 120 h (40%) (Figure 4A+4B+4C). To further confirm apoptosis, cleaved Poly (ADP-ribose) polymerase (PARP) as well as the initiator caspases CASPASE8, CASPASE9 and the central effector CASPASE3 were assessed upon *siPOLD1* transfection at 96 h. Consistent with the increased sub-G1 fraction of *ATR*^s/s^ cells, cleavage of PARP, CASPASE3 and CASPASE9 was observed selectively in *ATR*^s/s^ but not in parental cells (Figure 4D). In addition, caspase cascade activity was determined by CASPASE3-dependent cleavage of the fluorogenic CASPASE3-specific substrate Ac-DEVD-AMC 96 h after *siPOLD1*-transfection. POLD1-depleted *ATR*^s/s^ cells exhibited a 6-fold increase in DEVDase activity, corresponding to CASPASE3 activity, whereas no significant DEVDase activity was observed in parental cells (Figure 4E).

### Effects of combined POLD1- and ATR-depletion on H2AX phosphorylation

**Figure 5:**
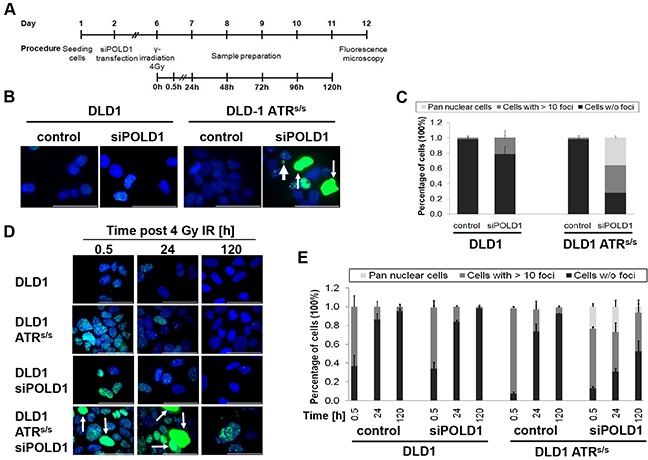
*ATR* and *POLD1* knockdown-dependent γ-H2AX staining Parental and *ATR^s/s^* cells were grown on coverslips, treated with *siPOLD1* at 10 nM or left untreated, then irradiated and stained with an anti-γ-H2AX antibody (green). Nuclei were counterstained with Hoechst 33258 (blue). **A.** Schematic representation of the experimental procedure. **B+D.** Representative images and **C+E.** γ-H2AX quantification of control versus *siPOLD1*-treated parental and *ATR^s/s^* cells, respectively, are shown **B+C.** at 120 h after transfection without irradiation and **D+E.** upon irradiation at 0.5 h, 24 h and 120 h. Thin arrows indicate pan-nuclear γ-H2AX staining, thick arrows apoptotic bodies. A scale bar (10 μm) is depicted. **C+D.** For quantification, at least 50 cells for each cell line and condition were scored in two independent experiments. Error bars represent SEM.

DNA damage- and DNA repair-kinetics were assessed upon *siPOLD1* transfection in parental and *ATR^s/s^* cells treated with either ionizing gamma-radiation (IR) or left untreated, using γ-H2AX intranuclear focus formation and elimination as well as pan-nuclear γ-H2AX staining as surrogate markers. In response to IR, intranuclear γ-H2AX foci as a marker for DNA double-strand breaks (DSBs) are rapidly formed within minutes, peak at 0.5 to 1 h and recover within 24 h [28, 29], while pan-nuclear γ-H2AX staining, displayed as diffuse staining of the whole nucleus, is restricted to S-phase-dependent replication stress [30, 31]. After verification of efficient siRNA-mediated *POLD1* knockdown at 96 h post transfection (Figure 2B), parental and *ATR^s/s^* cells were IR-treated at a previously determined sub-lethal dose of 4 Gy. Subsequently, γ-H2AX focus formation, elimination and pan-nuclear staining were quantified at multiple time points ranging from 0.5 to 120 h. The experimental set-up is schematically depicted in Figure 5A. Untreated parental and *ATR^s/s^* cells displayed no significant γ-H2AX focus formation or pan-nuclear staining. Upon *POLD1* knockdown, a fraction of parental cells exhibited increased γ-H2AX focus formation (21% of cells showing >10 foci/cell), while no significant pan-nuclear staining was observed. In contrast, *ATR^s/s^* cells displayed a large fraction of cells that exhibited either an increased γ-H2AX focus formation (36% of cells showing >10 foci/cell) or high levels of pan-nuclear staining (36% of cells) upon *POLD1* knockdown (Figure 5B+5C). Upon treatment with IR, a large fraction of γ-H2AX foci-positive cells was expectedly observed at 0.5 h for control (63% of cells showing >10 foci/cell) and POLD1-depleted parental cells (65%) and an even higher fraction for control and POLD1-depleted *ATR^s/s^* cells (approximately 90%), which is consistent with the known radio-sensitizing effects of ATR-deficiency [32]. However, POLD1-depleted *ATR^s/s^* cells additionally exhibited an increased fraction of H2AX-positive cells also at 24 h and even at 120 h after IR, including cells with increased γ-H2AX focus formation (63% at 24 h / 41% at 120 h), pan-nuclear staining (23% at 24 h /7% at 120 h), along with apoptotic body formation, indicating sustained DNA damage and/or impaired DNA-repair specifically in cells with combined *ATR*- and *POLD1*-defects as compared to control cells and cells harboring only one of these defects (Figure 5D+5E).

## DISCUSSION

In response to DNA damage and replication stress, ATR acts as central checkpoint regulator and mediator of DNA-repair by homologous recombination [12]. ATR-inhibition has recently been demonstrated to induce a selective elimination of certain subsets of tumor cells [14, 15] but the underlying genetic determinants are still insufficiently defined. Using a well-defined genetic *ATR* knock-in model of human CRC cells [23], we conducted a siRNA library screening approach to identify potential synthetically lethal interactions between *ATR* and certain DNA-repair genes. We identified six DNA-repair genes exhibiting synthetically lethal interactions with *ATR* and 20 genes displaying *ATR* genotype-independent knockdown-induced cell killing. Among the identified genes exhibiting synthetically lethal interactions with *ATR*, the most profound effects were observed for *POLD1* and further characterized.

*ATR* is an essential gene [33] and consequently, few cellular models exist to investigate its complete disruption. However, the bi-allelic hypomorphic *ATR* splice site mutation 2101^A→G^, naturally found in Seckel syndrome patients [34], results in subtotal ATR protein depletion without significant effects on cancer cell growth or viability [14, 22–24]. The human CRC line DLD1 engineered to homozygously harbor this mutation (termed *ATR^s/s^* cells) [14, 22–24] thus represents an ideally-suited model system for our question, as subtotal ATR protein depletion likely mimics the incomplete inhibition of ATR achievable through pharmacological means more closely than the complete and in most instances lethal *ATR* gene knockout [33]. Preliminary experiments confirmed that DLD1 *ATR^s/s^* cells display suppression of ATR protein below the detection limit of our assay as well as increased sensitivity towards MMC, as previously described [23, 24].

In our screen, we identified 26 DNA-repair genes, whose knockdown elicited either selective *ATR* genotype-dependent or -independent detrimental effects. Hit rates did not systematically differ between *ATR^s/s^* and *ATR*-proficient cells (hit rate = 9%), ruling out the systematic error of general siRNA-transfection-mediated cell killing of *ATR*-deficient cells. In addition, the screening validity was confirmed by a z factor of >0.5 [35]. The sensitivity of our approach was illustrated by the correct re-identification of the previously described synthetically lethal interactions of *XRCC1* or *PRIM1* with *ATR* [17, 19]. In addition, very recent data published during the writing of this manuscript retrieved some of the hits obtained in our genetic *ATR* model, including especially *POLD1* and *PRIM1*, in a less *ATR*-specific synthetic lethal screen using ATR-inhibitors [36].

We identified six DNA-repair genes, whose knockdown led to proliferation inhibition selectively of *ATR^s/s^* but not of *ATR*-proficient cells (hit rate = 2%). In addition, we found 20 genes, whose knockdown led to proliferation inhibition independently of *ATR* status (hit rate = 7%), indicating essential functions of these genes at least in DLD1 cells. The strongest *ATR* genotype-independent effects were observed for *XAB2* and *PLK1* knockdown, both of which resulted in a virtually complete proliferation loss. Consistently, homozygous *XAB2* and *PLK1* knockout mice display an early embryonic lethal phenotype [26, 27] and knockdown of *XAB2* was reported to induce widespread cell death in human bladder, cervix and pancreatic cancer [37].

The strongest effects selectively on DLD1 *ATR^s/s^* cells were observed for *POLD1* and *PRIM1* knockdown, both of which are involved in DNA replication synthesis [38, 39]. *POLD1* was further characterized as described below. *PRIM1* is the catalytic subunit of DNA primase synthesizing short RNA primers, which are extended in complex with DNA polymerase α [40]. A polymerase switch to DNA polymerase δ harboring the catalytic and proofreading subunit POLD1 ensures primer elongation and DNA strand polymerization. Accordingly, both proteins, PRIM1 and POLD1, are involved in immediately consecutive DNA replication steps [41], explaining the synthetically lethal effects upon depletion of either protein in *ATR*-deficient cells. Mechanistically, RNA primer synthesis influences replication-dependent binding of ATR to chromatin, which is required for checkpoint activation. Upon completion of DNA replication, dissociation of ATR from DNA triggers entry into mitosis [42]. Impairment of either PRIM1 or POLD1 in combination with ATR impairment might thus be expected to cause first incomplete DNA replication, which is then followed by premature entry into mitosis due to checkpoint deficiency.

In addition to *POLD1* and *PRIM1*, we identified *XRCC5* (*Ku80*) and *XRCC6* (*Ku70*) knockdown-induced proliferation inhibition of *ATR^s/s^* cells. Next to the role of XRCC5 and XRCC6 in non-homologous end joining DNA repair [43], the XRCC5/XRCC6 heterodimer complex associates with the essential factors MCM [44] and ORC [45] to form the pre-replication complex. Consistently, low expression levels of *XRCC6* and *XRCC5* lead to decreased DNA synthesis due to abortive DNA replication initiation [46], which in combination with impaired ATR-mediated checkpoint signaling might be expected to cause synthetic lethality between *ATR* and *XRCC5/XRCC6* through a similar mechanism as explained above. Clinically, *XRCC5* and *XRCC6* single nucleotide polymorphisms as well as epigenetic silencing of these genes can lead to the development of multiple cancers, such as CRC, breast and lung cancer [47]. It will be interesting to investigate in future studies, whether *XRCC5/XRCC6-*impaired tumors were sensitive towards ATR- or CHK1-inhibitors.

Clearly, additional studies are required to confirm and mechanistically characterize the synthetic lethal interactions between *ATR* and the genes identified in our study. As a start, we picked *POLD1* for in-depth characterization, as its knockdown elicited by far the strongest effects in *ATR^s/s^* cells. After confirmation of time- and *siPOLD1*-concentration-dependent cell killing specifically of *ATR^s/s^* cells, we demonstrated these effects to be reproducible pharmacologically by using chemical ATR-inhibitors on POLD1-depleted cells. Importantly, a general hypersensitivity phenotype of POLD1-depleted cells was excluded by treatment with various chemotherapeutics including ICL- and non-ICL-agents, none of which elicited *POLD1*-dependent hypersensitivity.

Intracellular protection against DNA damage and replication stress is mediated by both ATR and its downstream major effector kinase CHK1. Both proteins are essential and appear to similarly promote tumorigenesis [33, 48, 49]. As CHK1-inhibitors are currently further developed than ATR-inhibitors [13] and already undergoing testing in clinical trials [50], we asked, whether the effects of ATR-inhibition could similarly be induced by CHK1-inhibition. We applied the CHK1-inhibitor UCN-01 for this purpose despite its rather low selectivity, because it currently represents the only FDA-approved CHK1-inhibitor [50]. Importantly, UCN-01 caused comparable effects on POLD1-depleted cells as did ATR-inhibitors. Nevertheless, *ATR* and *CHK1* have been demonstrated to not function completely epistatically [51] and consequently, ATR- and CHK1-inhibitors are not expected to be readily exchangeable. Besides the canonical phosphorylation of CHK1 by ATR, multiple other substrates are phosphorylated by ATR in various tumor identities [11, 14, 52]. Vice versa, kinases other than ATR have been demonstrated to mediate compensatory ATR-independent CHK1 activation [53]. Consequently, inhibition of ATR as the upstream kinase of CHK1 is expected to elicit additional and at least partly distinct effects than CHK1-inhibition when applied for cancer-therapeutic approaches.

In an effort to generalize our data beyond one single cell line, we investigated the effects of ATR- and CHK1-inhibitors in a panel of CRC cell lines, including lines exhibiting a microsatellite instable (MSI) as well as those exhibiting a chromosomal instable (CIN) phenotype [54, 55]. POLD1-depleted RKO, SW480 and LS513 cells all displayed increased sensitivity towards ATR-/CHK1-inhibitors as compared to control cells. The fact that only some but not all ATR-/CHK1-inhibitors elicited *POLD1-*dependent effects might be ascribable to the additional unspecific inhibition of other targets inherent to chemical inhibitors along with the heterogeneous genotype of the tested CRC lines. Nevertheless, inhibition of the ATR/CHK1-axis could be a generalizable therapeutic concept in patients with *POLD1* low-or non-expressing tumors.

To investigate the underlying mechanism of the synthetic lethal interaction between *ATR* and *POLD1*, we analyzed cell cycle distribution to detect cell cycle arrests along with the sub-G1 fraction as a surrogate marker for apoptosis. While no significant effects on cell cycle were observed, we found a significantly increased sub-G1 fraction in *ATR^s/s^* cells upon *POLD1* knockdown. Apoptosis was further confirmed by the proteolytic cleavages of PARP, the initiator CASPASE9 and the executioner CASPASE3 [56] as well as by CASPASE3-attributable DEVDase activity [57]. In general, these data are consistent with previous studies showing spontaneous apoptosis *in vivo* in *POLD1^−/−^* mice [58]. More specifically, *POLD1* downregulation has been demonstrated to mediate the reduction of DNA synthesis *in vitro* [59], which is expected to activate the DNA replication checkpoint [60]. Disruption of this checkpoint by ATR deficiency might thus prevent cell cycle arrest in S-phase, a hypothesis supported by the absence of cell cycle disturbances in our experiments. Taken together, reduction of DNA synthesis caused by *POLD1* knockdown along with premature entry into mitosis caused by *ATR* deficiency provides a plausible mechanism for the apoptosis-mediated synthetic lethality of *POLD1* and *ATR* in our experiments.

Since POLD1 represents a DNA polymerase δ subunit with critical catalytic and proofreading activity in replicative DNA synthesis, recombination and especially repair processes [38], we investigated the effects of POLD1 depletion on DNA damage- and DNA repair-kinetics in *ATR*-proficient versus *ATR-*deficient cells. Upon *POLD1* knockdown, *ATR^s/s^* cells but not parental cells displayed strongly increased levels of endogenous DNA DSBs, as illustrated by increased nuclear γ-H2AX focus formation [61]. Upon exogenously induced DNA DSBs by IR, sustained γ-H2AX focus accumulation (>120 h) was observed specifically in *siPOLD1*-transfected *ATR^s/s^* cells, but not in untransfected *ATR^s/s^* cells or untransfected or transfected parental cells, strongly supporting an impaired or at least decelerated DNA-repair capacity. These data further support our above hypothesis that depletion of *POLD1* causing increased DNA-damage [59] and decreased DNA-repair in combination with deficient *ATR*-signaling causing DNA replication checkpoint disruption [60], premature entry into mitosis and eventually apoptosis mechanistically explains the synthetic lethality of these two genes.

Importantly, previously reported genome-sequencing data put our study in a direct clinical context. A missense mutation (p.His506Arg) in the exonuclease domain III of DNA polymerase δ, expected to cause a hypermutability phenotype, has earlier been identified in human CRC lines [62]. In addition, recently described *POLD1* missense mutations predispose to CRC (p.Ser478Asn, p.Pro327Arg), endometrial cancer (p.Ser478Asn) and likely to brain (p.Ser478Asn) and kidney tumors (p.Val392Met) [63, 64]. Equivalent mutations of the human *POLD1* p.Ser478Arg lead to an increased mutation rate in fission yeast and are mapped along with the human *POLD1* p.Pro327Arg mutation at the interface of the exonuclease active site, predicting these mutations to have functional effects on DNA-binding and exonuclease activity [64]. Thus, functional genetic alterations of *POLD1* could represent predictive markers for therapeutic response towards ATR- and CHK1-inhibitors in the clinical setting. However, regarding colorectal cancer, at least 12 known CRC cell lines have been reported to harbor either heterozygous or homozygous mutations in *POLD1* [65]. As many of these mutations represent variants of unknown significance, future studies applying suitable syngeneic *POLD1* model systems are urgently needed to clarify the functional significance of these genetic changes in colorectal cancer as well as other tumor entities.

In conclusion, ATR-inhibition induces the selective elimination of certain cancer cell subsets [14, 15], but the underlying genetic determinants remained insufficiently defined. By screening of a DNA-repair gene siRNA library in an *ATR* cancer cell model, we identified *POLD1* as one critical determinant during ATR inhibition-mediated CRC cell killing. Currently ongoing whole-genome sequencing studies are expected to additionally determine the *POLD1* mutation rates in tumor entities other than CRC or endometrial cancer, which could then broaden the applicability of the here proposed concept of a novel tumor genotype-based anti-cancer therapy.

## MATERIALS AND METHODS

### Cell lines and culture conditions

The human CRC cell lines DLD1, RKO, SW480 and LS513 were purchased from the European Collection of Cell Cultures (Sigma-Aldrich, Munich, Germany) or the American Type Culture Collection (LGC Standards, Wesel, Germany), respectively. DLD1 cells homozygously harboring the hypomorphic Seckel mutation (*ATR^s/s^*) have been described previously [14, 23, 24]. This mutation causes a strongly reduced but not absent *ATR* protein expression without significant impairment of cell proliferation or survival [24]. All cell lines were maintained in Dulbecco's modified Eagle's medium (DMEM) supplemented with 10% fetal calf serum and 1% penicillin-streptomycin (PAA, Coelbe, Germany) and incubated at 37°C and 5% CO_2._

### Reagents

MMC and UCN-01 were purchased from Sigma-Aldrich (Steinheim, Germany), LY2603618 from Selleckchem (Munich, Germany), NU6027 from Merck (Darmstadt, Germany), VE-822 from MedKoo Bioscience (Chapel Hill, NC, USA), 5-FU from Medac (Wedel, Germany), and oxaliplatin from Accord Healthcare (Freilassing, Germany).

### siRNA library screening

A siRNA library was used containing 288 validated DNA-repair genes each targeted by 3 validated siRNAs (QIAGEN, Hilden, Germany). 800 to 1,000 cells/well were seeded in 96-well plates to reach confluence at day 7. 24 h later, transfection was performed in supplementary-free medium with the respective siRNAs or no siRNA at a final concentration of 10 nM using Oligofectamine (Invitrogen, Darmstadt, Germany) in OptiMEM (Gibco, Life Technologies GmbH, Darmstadt, Germany). 4 h after transfection, serum-containing medium was added to the cells. 120 h after transfection, cells were washed, lysed in 100 μL H_2_O, and 0.2% SYBR®Green (Lonza, Cologne, Germany) was added. Fluorescence was measured using a CytoFluor Series 4000 plate reader (PerseptiveBiosystems, Framingham, MA, USA). Four independent siRNA library screens were performed with each siRNA data point reflecting triplicate wells. The growth inhibition was determined by dividing each siRNA-treated value by the average of 12 untreated control values for both parental DLD1 and DLD1 *ATR^s/s^* cells. The growth inhibition ratio was calculated by dividing the growth inhibition value of parental DLD1 by the value of *ATR^s/s^* cells. The mean growth inhibition ratio and the standard error of the mean (SEM) were determined from four individual growth inhibition ratio values that each represented triplicates from three different oligonucleotides targeting one particular gene. DNA-repair genes were classified into hit categories defined as either “selective *ATR* genotype-dependent” or “*ATR* genotype-independent” proliferation inhibition. DNA-repair genes were scored as “selective *ATR* genotype-dependent” hits if the mean growth inhibition ratio was >1.50 and the average relative survival of parental DLD1 cells was >0.45. Gene targets causing comparable growth inhibitions in parental and *ATR^s/s^* cells were scored as “*ATR* genotype-independent” hits. The average relative survival of parental and *ATR^s/s^* cells, respectively, was calculated by the mean of four individual growth inhibition values for each cell line from three different oligonucleotides targeting one particular gene. As preliminary experiments confirmed no relevant proliferation differences between untreated and mock-treated cells, untreated cells were used as controls in the following screening experiments.

### Individual *siPOLD1* transfection experiments

Cells at 30%-50% confluence were transfected in supplementary-free medium using Oligofectamine and siRNA directed against *POLD1* (QIAGEN) at final concentrations of 2.5, 5, 10, 20, 40, 50, 80 nM or a non-coding sequence of β-galactosidase (βGAL, Dharmacon Lafayette, Co, USA) at 50 nM or no siRNA (mock). Transfection proceeded for 4 h before adding serum-containing medium. The following siRNA sequences were used: *siPOLD1*-1 (*siPOLD1*) CGGGACCAGGGAGAATTAATA, *siPOLD1*-2 CAGTT GGAGATTGACCATTAT, *siPOLD1*-3 CCGAGAGAG CATGTTTGGGTA, si*β*GAL UUAUGCCGAUCGCGU CACAUU.

### Cell proliferation assays

Cell proliferation assays were performed over a broad range of concentrations covering 100% to 0% cell survival. 800 to 3,000 cells/well were plated in 96-well plates to reach confluence on day 7. After settling, the cells were incubated with various drugs at multiple concentrations. Following incubation for 120 h, the cells were washed, lysed in 100 μL H_2_O and 0.2% SYBR®Green was added. Fluorescence was measured using a CytoFluor Series 4000 plate reader and growth inhibition was calculated as compared to the untreated control samples. At least, three independent experiments were performed per drug, with each data point reflecting triplicate wells. Error bars represent SEM of three experiments, independently performed in triplicates.

### Immunoblotting

Cells were lysed and protein extracts boiled and loaded on 8% polyacrylamide gels. After electrophoresis, proteins were transferred to PVDF membranes, which were blocked for 1 h in 5% milk powder before primary antibody was applied at 4°C overnight. The membranes were washed and stained with secondary antibody. Enhanced chemo-luminescence was elicited using ECL Western Blotting Substrate (Thermo Scientific, Schwerte, Germany) according to the manufacturer's instructions. The following primary antibodies were used: anti-CASPASE3, anti-CASPASE8, anti-CASPASE9, anti-PARP (all Cell Signaling Technology, Boston, MA, USA); anti-POLD1 (sc-8797, Santa Cruz Biotechnology, Heidelberg, Germany). Anti-β-ACTIN antibody (Sigma-Aldrich) served as loading control. The following secondary antibodies were used: anti-goat HRP-conjugated antibody (Santa Cruz Biotechnology); anti-mouse and anti-rat HRP-conjugated antibody (GE Healthcare, Freiburg, Germany).

### Nuclear γ-H2AX focus formation assay

Cells were grown on coverslips in 6-well plates. At 60% confluence, the cells were irradiated at a dose of 4 Gy using a RS225 γ-ray tube (X-Strahl, Camberley, Great Britain). Consecutively, treated cells were washed, fixed for 10 min in 3.7% formaldehyde and for 1 min in methanol. After permeabilization in TBS/0.5% Triton X-100 and blocking in TBS/2% BSA/0.5% Triton X-100, cells were incubated with an anti-phospho- H2AX antibody (mouse monoclonal, Upstate Biotechnology Inc., NY, USA) for 2 h. Afterwards, the cells were washed and incubated with Alexa 488 goat anti-mouse antibody (Invitrogen) for 2 h. After washing, nuclei were counterstained with Hoechst 33258 (Sigma-Aldrich) at 10 μg/ml. Slides were mounted with VECTASHIELD mounting medium (Burlingame, CA, USA) and analyzed using a Zeiss AxioVision fluorescent microscopy (Carl-Zeiss, Jena, Germany) and the AxioVision Re.4.8 software (Carl-Zeiss). Exposure time and settings were kept constant for all samples in individual experiments. At least 50 cells were scored for each cell line and each condition, applying two independent experiments. Error bars represent SEM.

### Cell cycle analysis

Cells were grown in 6-well plates. At 30% confluence, the cells were transfected with *siPOLD1* at 10 nM or mock-treated. After 24, 48, 72, 96 and 120 h, cells were collected, washed and incubated in staining buffer (0.1% sodium citrate, 0.1% Triton X-100 and 50 μg/ml propidium iodide) according to the method by Nicoletti [66]. Quantification of cell cycle distribution and subG1-cell fraction were analyzed by flow cytometry (Accuri C6 Flow Cytometer^®^, BD Biosciences, San Jose, CA, USA) and CFlow Plus software (BD Biosciences). Per sample, 20.000 events were acquired. Error bars represent SEM of three experiments, independently performed in triplicates.

### Fluorometric assay for caspase activity

For detection of CASPASE3-like DEVDase activity, 800 to 1,000 cells/well were plated in 96-well plates to reach confluence on day 7. After settling, transfection was performed with siRNAs directed against *POLD1* at a final concentration of 10 nM. Following incubation of 96 h, the cells were prepared in lysis buffer containing 0.5% Nonidet P-40, 20 mM HEPES (pH 7.4), 84 mM KCl, 10 mM MgCl_2_, 0.2mM EDTA, 0.2 mM EGTA, 1 mM DTT, 5 g/ml aprotinin, 1 g/ml pepstatin, and 1 mM phenylmethylsulfonyl fluoride (PMSF). Caspase activity of 20 μg cell lysate was determined with 50 μM of the fluorogenic substrate Ac-DEVD-AMC (*N*-acetyl-Asp-Glu-Val-Asp-aminomethyl-coumarin, Biomol, Hamburg, Germany) as described before [57]. Concomitant treatment of cells with tumor necrosis factor alpha (TNFα) at 25 ng/ml and actinomycinD (AcD) at 200 ng/ml for 6 h was used as positive control [67, 68]. Error bars represent SEM of two experiments, independently performed in triplicates.

### Statistical analysis

All statistical analyses were performed using IBM SPSS Statistics 21 (SPSS Inc., Chicago, IL, USA). Error bars represent SEM of at least three experiments, except for the fluorometric assays for caspase activity and the γ-H2AX focus formation assays, which were performed twice. FACS data were statistically interpreted using a paired Student's t-test. P-values **p < 0.01 were considered statistically significant.

## SUPPLEMENTARY FIGURE


