# Supplementary material for: A synthetic lethal screen identifies ATR-inhibition as a novel therapeutic approach for POLD1-deficient cancers
Source: Oncotarget. 2016 Jan 9;7(6):7080–95. doi: 10.18632/oncotarget.6857 (PMC4872770; doi:10.18632/oncotarget.6857)
Supplement: Supplementary file 1 [file oncotarget-07-7080-s001.pdf]

SUPPLEMENTARY FIGURE

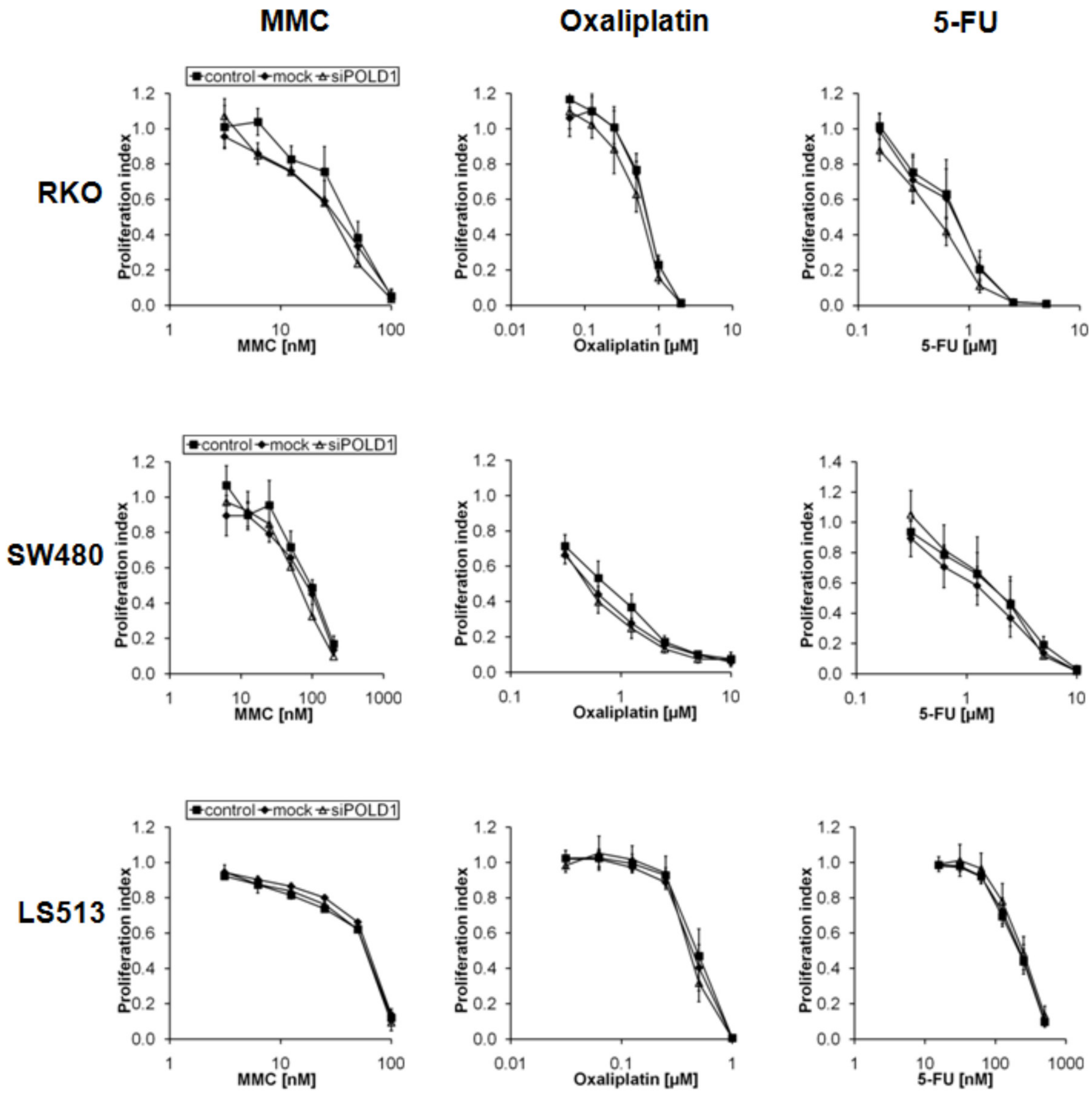

Supplementary Figure S1: Chemotherapy-induced proliferation inhibition upon *POLD1* knockdown in a panel of CRC cell lines. Effects on proliferation of MMC, oxaliplatin and 5-FU were assessed at 120 h after treatment in control-, mock- or *siPOLD1*-treated RKO, SW480 and LS513 cells. Error bars represent SEM of three independent experiments with each data point reflecting triplicate wells.
